# A Case Series of Deep Transcranial Magnetic Stimulation Treatment for Patients with Obsessive-Compulsive Disorder in the Tokyo Metropolitan Area

**DOI:** 10.3390/jcm11206133

**Published:** 2022-10-18

**Authors:** Haruki Ikawa, Ryota Osawa, Akiko Sato, Hoshimi Mizuno, Yoshihiro Noda

**Affiliations:** 1 Tokyo Yokohama TMS Clinic, Kawasaki 211-0063, Japan; 2 Department of Neuropsychiatry, Keio University School of Medicine, Tokyo 160-8582, Japan

**Keywords:** anterior cingulate cortex, deep transcranial magnetic stimulation, medial prefrontal cortex, neuromodulation, obsessive-convulsive disorder

## Abstract

Obsessive-compulsive disorder (OCD) is a chronic illness in which patients do not achieve remission sufficiently with conventional medication. Deep repetitive transcranial magnetic stimulation (dTMS) for OCD neuromodulates the bilateral anterior cingulate cortex (ACC) and dorsal medial prefrontal cortex (mPFC), which are known to be impaired in OCD. While dTMS treatment for OCD has shown effective results overseas, TMS treatment for OCD has rarely been implemented in Japan, and its effectiveness is unknown. We conducted an FDA-approved dTMS protocol to 26 patients with OCD. In addition, individual exposure stimulation that elicited each patient’s obsessive thoughts was also combined during dTMS treatment. Before and after 30 sessions of TMS treatment, the Yale-Brown Obsessive Compulsive Scale (Y-BOCS) was used to assess changes in the severity of each patient’s obsessive-compulsive disorder. Response to dTMS treatment in patients with OCD was determined by whether the total score on the Y-BOCS after a course of treatment was reduced by 30% or more compared with the score at baseline. The percentage of responders in this case series following the 30 sessions of dTMS treatment was 53.9%. In addition, total Y-BOCS scores and scores on each item were significantly improved. The percent changes in total Y-BOCS scores did not differ between the sexes or between on- and off-medication patients. No obvious adverse events were observed in this case series. In line with the results of TMS studies for OCD patients reported overseas, dTMS treatment for Japanese patients with OCD may have a favorable therapeutic effect.

## 1. Introduction

Obsessive-compulsive disorder (OCD) is a chronic illness and is known for a lifetime prevalence of 2–3% [1]. OCD is characterized by intrusive and persistent thoughts that provoke anxiety, distress (obsessions), and habitual behaviors that could neutralize obsessions (compulsions) [2]. More than 30% of patients with OCD do not achieve remission with conventional medication or cognitive behavioral therapy (CBT) [3]. In this context, the development of novel and effective therapeutic strategies for OCD is expected. Previous neuroimaging studies on OCD have shown that the anterior cingulate cortex (ACC) and medial prefrontal cortex (mPFC) are impaired [4,5]. The ACC and mPFC are considered part of the limbic network that regulates the frontal basal ganglia circuit and is associated with a shift between goal-directed (obsessions lead to subsequent changes in compulsions) and habitual behaviors [6,7]. The original version of deep repetitive transcranial magnetic stimulation (dTMS) for OCD uses Brainway’s H7-coil to directly stimulate the bilateral dorsal mPFC and ACC, causing neurons in the same region to fire, thereby altering neuroplasticity in those regions and modulating the frontal-basal ganglia circuit [8]. Indeed, in 2018, the U.S. Food and Drug Administration (FDA) approved neuromodulation therapy for treatment-resistant OCD with high-frequency dTMS targeting the bilateral dorsal mPFC and ACC using H7-coils based on the positive results from a multicenter sham-controlled trial [9]. In the same study, obsessive-compulsive symptoms were assessed with the Yale-Brown Obsessive Compulsive Scale (Y-BOCS) to evaluate clinical efficacy, and response rate was defined as a 30% decrease in the Y-BOCS score compared with the baseline [9,10]. The response rate of patients treated with active dTMS (38.1%) was significantly higher than that of patients treated with sham stimulation (11.1%).

However, in Japan, dTMS treatment for OCD has not yet been approved by public national health insurance and has rarely been implemented either in private practice or in clinical studies. Thus, the efficacy of dTMS treatment for OCD is not clear in the Japanese population. With this background, the objective of this case series was to examine the clinical outcome of a total of 30 sessions of dTMS for patients with OCD at a TMS clinic in the Tokyo metropolitan area on a real-world research basis.

## 2. Materials and Methods

### 2.1. Participants

The present case series study included 26 patients with OCD (14 males, 12 females, mean age 35.8 ± 10.9, 14/26 on medication) (Table 1) who were outpatients at the Tokyo Yokohama TMS Clinic. In addition, medication and clinical information of the included patients were summarized in Table 2. Note that all cases included in this case series were only those who had been diagnosed with OCD at an outside psychiatric institution and were also confirmed as OCD by psychiatrists (Y.T., H.I., and R.O.) based on the DSM-5 at the time of their first visit to this TMS clinic.

### 2.2. dTMS Protocol

**Figure 1:**
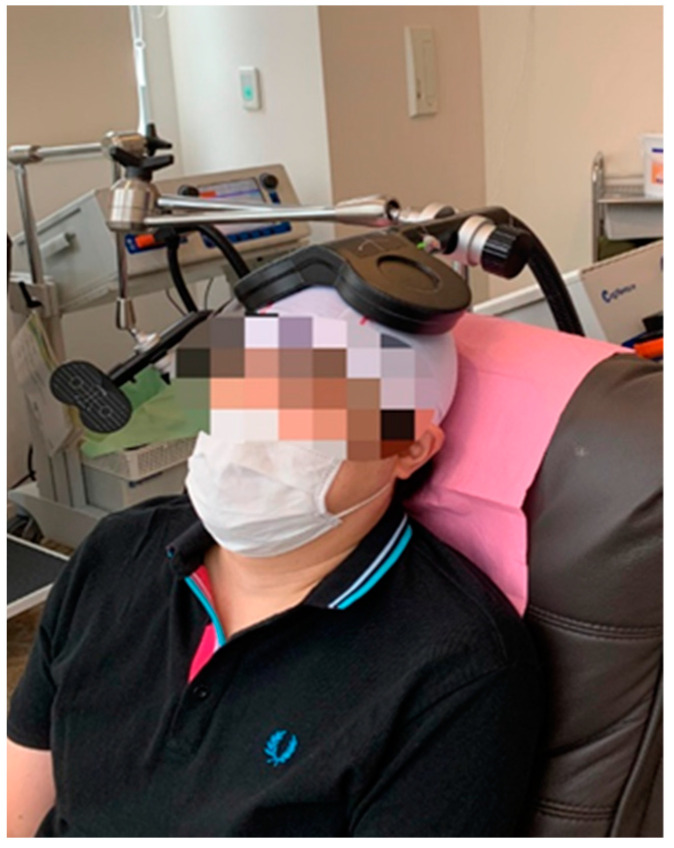
The photo shows the placement of the butterfly coil with the patient’s bilateral dorsal mPFC as the stimulation target in this treatment protocol.

The study used the U.S. FDA-approved dTMS protocol targeting the bilateral dorsal mPFC; specifically, a total of 30 sessions of 2000 pulses at 20 Hz, 2-s pulse trains, 20-s intertrain intervals, and 50 trains per session at 100% resting foot motor threshold [9]. In this case series, a total of 30 dTMS treatment sessions were conducted over a 10-week period at a frequency of 3 times per week. Furthermore, we used the Cool D-B80 Butterfly, Active cooling coil (MagVenture, Inc., Farum, Denmark) in this study. To stimulate the bilateral dorsal mPFC with the butterfly coil, the coil was placed 4 cm anterior (see Figure 1) to the area innervating the dorsal foot in the motor cortex [9].

### 2.3. Resting Motor Threshold (RMT) Measurement

In this case series, electromyography was not used for RMT measurement, but visual confirmation was used. RMT was defined as the intensity at which muscle contraction of the anterior tibial muscle was visually confirmed at least 5 times out of 10 stimulations of the TMS.

### 2.4. Individualized Exposure Stimulation

In this study, we also combined individualized exposure stimulation that provokes obsessions in each patient during dTMS treatment. Specifically, immediately prior to the introduction of the initial dTMS treatment, each patient was interviewed to create an anxiety hierarchy chart from level 1 to 3 regarding the situations causing obsessive-compulsive symptoms. During the dTMS sessions, each patient was first asked to imagine an anxiety level 1 situation while being treated. Thereafter, the anxiety level was gradually increased in discussion with the patient, which was in line with the treatment protocol described in the literature [9].

### 2.5. Clinical Assessment

Patients with OCD underwent clinical assessment with the Y-BOCS before and after 30 treatment sessions. Moreover, all patients agreed to participate in the TMS database registry study (jRCT1050210059) [10]. The response of patients with OCD to dTMS treatment was determined by whether the total score on the Y-BOCS decreased by 30% or more compared to baseline, based on the definition of previous studies [11]. In this case series, a paired *t*-test was performed to compare the baseline score before dTMS treatment and the score on the Y-BOCS after the 30 sessions course of dTMS treatment.

### 2.6. Sub-Analysis Based on Clinical Epidemiological Information

Furthermore, sub-analyses were performed to examine whether there was any sex or medication status difference with respect to the response rate as indexed by the percent changes in the total score on the Y-BOCS following dTMS treatment. 

### 2.7. Statistical Analysis

We used paired *t*-tests to compare the pre-post clinical outcome as measured by the Y-BOCS following dTMS. Since this study was an open-label pilot case series, it was based on the naive hypothesis that dTMS treatment combined with individualized exposure stimulation for OCD patients might reduce Y-BOCS scores. Thus, in this analysis, the significance level was set at 0.01 with a two-tailed test. In addition, in relation to the subanalysis in 2.6 above, we conducted a logistic regression analysis using response defined by Y-BOCS as the dependent variable and sex and medication status as the independent variables to examine whether these independent variables can significantly explain treatment response to dTMS treatment in patients with OCD.

### 2.8. Assessment of Adverse Events

Of note, we assessed the adverse events and side effects that could be associated with this dTMS protocol combined with exposure stimulation in this case series.

## 3. Results

**Figure 2:**
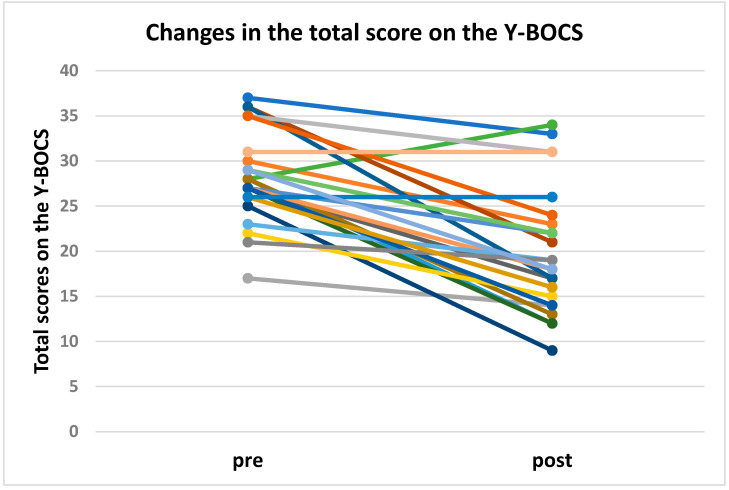
Changes in the total score on the Y-BOCS in patients with OCD following dTMS treatment combined with individualized exposure stimulation. Before and after a course of treatment, the mean scores (±S.D.) on the Y-BOCS of patients with OCD improved from 28.5 (5.0) to 20.0 (6.7). Each color in the line graph corresponds to a different case. The breakdown indicated that 23 of the 26 patients showed improvement in scores, 2 remained unchanged, and 1 worsened.

Clinico-demographic data at baseline is summarized in Table 1 and Table 2. The mean (±standard deviation (S.D.)) value of RMT in this case series was 56.0 ± 7.4%. The severity of OCD in patients included in this case series was considered severe, with a mean Y-BOCS total score of 28.5 (±5.0). Furthermore, the percentage of responders in this case series who improved their Y-BOCS scores by 30% or more following the 30 sessions of dTMS treatment was 53.9% (t_25_ = 7.12, *p* < 0.01) (Table 3 and Figure 2). Significant improvements in each of the Y-BOCS items are as follows: “time occupied by obsession”, “interference due to obsessive thoughts”, “distress associated with obsessions”, “control over obsessions”, “time spent on compulsions”, “distress associated with compulsions”, “resistance against compulsions”, and “control over compulsions”. We summarized the results of *t*-tests for the mean reduction of each score in the Y-BOCS (%) between males and females on- and off-medication in Table 4. The percent changes in total Y-BOCS scores did not differ between the sexes or between on- and off-medication patients. Furthermore, the logistic regression analysis revealed that either sex or medication status did not significantly affect the response to dTMS treatment in patients with OCD in this case series (summary of the model: Nagelkerke R2 = 0.000; B = 0.154, S.E. = 0.393, Wald = 0.154, *p* = 0.695, Exp(B) = 1.167; sex: *p* = 0.225, medication: *p* = 0.671).

Moreover, no obvious adverse events were observed in this case series. As a general side effect, 2 out of 26 patients complained of stimulation site pain during the dTMS session.

## 4. Discussion

To the best of our knowledge, this is the first case series report in Japan to demonstrate the results of dTMS treatment for patients with OCD. The response rate of 53.9% for this case series was comparable to the response rate of 57.9% in a previous real-world collaborative study of 29 sessions of dTMS conducted at 22 clinical sites in the United States, Israel, and Turkey [12]. On the other hand, dTMS treatment for OCD in a multicenter, randomized, double-blind, placebo-controlled trial reported a response rate of 38.1% [9]. With respect to the relatively low response rate in that randomized controlled trial (RCT) for OCD, Roth et al. interpreted that the uncertainty of whether participants were in the active treatment arm or the sham stimulation arm may have caused anxiety about treatment efficacy, resulting in a reduced treatment effect [12,13]. In addition, in the present case series, exposure stimulation that provoked obsessions was also combined during dTMS treatment, which may have augmented the treatment effects.

There were significant reductions in the total score on the Y-BOCS and the subscores on the Y-BOCS (“time occupied by obsession”, “interference due to obsessive thoughts”, “distress associated with obsessions”, “control over obsessions”, “time spent on compulsions”, “distress associated with compulsions”, “resistance against compulsions”, “control over compulsions”). Here, as a possible therapeutic mechanism to explain these results, dTMS targeting the bilateral dorsal mPFC may enhance the ability to control obsessions, as a direct effect on the cortex and may also indirectly suppress the runaway OCD circuits centered in the frontal lobes and basal ganglia that automatically generate obsessions and compulsive behaviors [6,8]. On the other hand, however, there was no significant decrease in the following items of the Y-BOCS (“resistance to obsessions” and “interference by compulsions”). Kim et al. reported that patients with OCD who had strong “resistance” factors (“resistance to obsessions” and “resistance to compulsive behavior”) were more difficult to treat [14]. Thus, when “resistance to compulsions” is too strong, treatment may be quite difficult even with dTMS treatment. Furthermore, Laposa et al. noted that in CBT for OCD, a change in obsessions leads to a subsequent change in compulsions [15]. This is in line with our findings that “interference due to compulsions” does not change immediately and significantly, due to the time lag between therapeutic changes in obsessions and compulsions.

On the other hand, there was no significant difference in the mean percent reduction in the total score on the Y-BOCS between males and females or between on- and off-medication. These results were consistent with the previous study that showed no difference in the response rate between males and females or between on- and off-medication [16].

In this case series, 23 of 26 patients with OCD showed improvement in their Y-BOCS scores, two remained unchanged, and one had a worsening score. The case with the worsening score appear to have temporarily worsened obsessive-compulsive symptoms in response to increased psychosocial stress related to the individual’s work, rather than a worsening of symptoms associated with dTMS treatment. In addition, two participants claimed mild headache (i.e., pain at the stimulation site) during dTMS session as a side effect. However, no serious adverse events occurred. This is in line with the results of a previous review [17].

Note that, to date, various stimulation protocols have been implemented in dTMS treatment for patients with OCD and that a network meta-analysis by Fitzsimmons et al. [18] revealed that (1) low frequency rTMS to the pre-supplementary motor area, (2) high frequency rTMS to the bilateral dorsolateral prefrontal cortex, and (3) low frequency rTMS to the right dorsolateral prefrontal cortex were more effective than dTMS to the bilateral dorsal mPFC, which was used in this study. In addition, Liang et al. also reported similar results in their meta-analysis, while reporting that there was no significant difference in the tolerability of each stimulation protocol between any of the stimulation methods [19].

Moreover, Cocchi et al. reported that dTMS could be more likely to elicit therapeutic effects when the subject was exposed to the stimulus that provoked the obsessive-compulsive symptoms than when the subject was in a resting state [20]. Thus, in this case series, dTMS treatment targeting the bilateral dorsal mPFC was used to suppress overactivation of the ACC in patients with OCD, which is known to be increased by exposure to obsessive-compulsive symptom-inducing imagery in this disorder. There are some limitations in this study. First, the sample size was small, which could be subject to type II errors in the statistical analysis. Second, due to the nature of an open-label case series, this study report has not been rigorously validated with an RCT design. Thus, the results warrant further validation in RCTs with larger sample sizes. Third, in this case series, since there was no follow-up evaluation by Y-BOCS after completion of dTMS treatment, it is unclear to what extent the therapeutic effect is maintained at, for example, 3, 6, and 12 months. Hence, systematic clinical studies with a RCT design and follow-up evaluation for a certain period of time are needed in the future. Fourth, this study did not use an MRI-guided navigation system that could more accurately identify the target brain region. In addition, since the RMT is essentially determined by recording the myoelectric activity of the target muscle, the RMT in this study may have been slightly higher than when the navigation system was used. Fifth, although no obvious adverse events were observed in this case series, a common side effect was that two of the 26 patients complained of pain at the stimulation site during the dTMS session. This could also have been induced by the relatively high stimulation intensity settings due to the limitations of the aforementioned methodology.

## 5. Conclusions

We conducted a total of 30 sessions of dTMS treatment combined with individualized exposure stimulation for 26 patients with OCD on a real-world setting. As reported in previous dTMS studies for OCD, dTMS treatment in Japanese patients with OCD was found to have a favorable therapeutic effect. Large-scale collaborative RCT studies using novel dTMS protocols for patients with OCD in Japan and elucidation of their therapeutic mechanisms are also awaited.

## Figures and Tables

**Table 1:** Clinico-demographic data of patients with OCD at baseline.

Sample Size		26	
Age (mean ± S.D.)		35.8 (12)	
Sex	Male	14	54
	Female	12	46
%patients on medication		14	53.9%

**Table 2:** Medication and Clinical information of included patients.

Case	Medication Treatment	Duration of OCD (Years)	Duration of Medication (Years)	Comorbidity
1	Paroxetine CR 50 mg, Mirtazapine 15 mg, Clonazepam 2 mg, Cloxazolam 1 mg	25	Details unknown	-
2	Lexapro 10 mg	4	4	-
3	Maprotiline 100 mg, Vortioxetine 20 mg	30	3	MDD
4	-	20	0	MDD
5	-	1	1	-
6	Paroxetine CR 25 mg, Alprazolam 0.4 mg	9	1	-
7	-	12	0	MDD
8	-	6	0	-
9	Fluvoxamine 150 mg, Alprazolam 0.8 mg, Risperidone 1 mg, Lithium 1000 mg, Brotizolam 0.25 mg	Details unknown	Details unknown	BD
10	Paroxetine CR 12.5 mg, Aripiprazole 3 mg	4	1	-
11	Lamotrigine 150 mg, Valproate 100 mg, Bromazepam 5 mg	1	1	MDD
12	Paroxetine 25 mg	9	1	ADHD
13	-	1	0	-
14	-	1	0	-
15	-	33	Details unknown	-
16	Lexapro 10 mg, Brexpiprazole 0.5 mg, Mirtazapine 15 mg	4	4	-
17	-	19	17	SAD
18	Paroxetine 45 mg, Lithium 600 mg	Details unknown	Details unknown	BD
19	Fluvoxamine 150 mg	13	1	-
20	-	Details unknown	Details unknown	-
21	-	11	3	MDD
22	Fluvoxamine 300 mg, Risperidone1 mg	1	1	-
23	-	1	0	-
24	-	10	Details unknown	MDD
25	Paroxetine 50 mg, Zopiclone 10 mg	47	10	-
26	Clomipramine 75 mg, Fluvoxamine 100 mg, Bromazepam 15 mg	27	27	MDD

MDD: major depressive disorder; BD: bipolar disorder; ADHD: attention deficit hyperactivity disorder; SAD: social anxiety disorder.

**Table 3:** Changes in individual items of the Y-BOCS following dTMS treatment combined with individualized exposure stimulation.

	Mean (S.D.)		*t*-Value	*p*-Value
	Pre-dTMS	Post-dTMS		
Total score of the Y-BOCS	28.5 (5.0)	20. (6.67)	7.12	* *p* < 0.01
Individual items on the Y-BOCS factors				
1. Time occupied by obsession	3.0 (1.0)	2.2 (0.8)	3.98	* *p* < 0.01
2. Interference due to obsessive thoughts	2.8 (0.8)	2.1 (0.7)	3.94	* *p* < 0.01
3. Distress associated with obsessions	3.3 (0.8)	2.4 (0.8)	5.12	* *p* < 0.01
4. Resistance against obsessions	2.5 (1.4)	1.7 (1.3)	2.27	*p* = 0.32
5. Control over obsessions	3.3 (0.9)	2.4 (0.9)	4.67	* *p* < 0.01
6. Time spent on compulsions	2.4 (0.8)	1.8 (0.9)	3.89	* *p* < 0.01
7. Interference due to compulsions	2.5 (0.7)	1.9 (0.9)	3.56	*p* = 0.02
8. Distress associated with compulsions	3.3 (0.9)	2.5 (0.9)	4.54	* *p* < 0.01
9. Resistance against compulsions	2.5 (1.3)	1.4 (1.2)	3.58	* *p* < 0.01
10. Control over compulsions	3.1 (0.8)	2.3 (1.0)	4.39	* *p* < 0.01
Responder (improvement of >30%)		14/26 = 53.9%		

* Asterisks were marked for significant findings that exceeded the significance level of 0.01 that was set for this study.

**Table 4:** Percent changes of the total score on the Y-BOCS (whole, male vs. female, and on-medication vs. off-medication) following dTMS treatment combined with individualized exposure stimulation.

		Whole	Males	Females
% changes of the Y-BOCS score	mean (S.D.)	29.8 (4.0)	24.9 (24.6)	35.5 (13.4)
	*p*-value	-	*p* = 0.20	
			On-medication	Off-medication
	mean (S.D.)	29.8 (4.0)	26.5 (23.3)	33.5 (17.1)
	*p*-value	-	*p* = 0.40	

## Data Availability

All clinical data are available upon reasonable request to the corresponding author.
